# Spatial variations of pulmonary tuberculosis prevalence co-impacted by socio-economic and geographic factors in People’s Republic of China, 2010

**DOI:** 10.1186/1471-2458-14-257

**Published:** 2014-03-17

**Authors:** Xin-Xu Li, Li-Xia Wang, Hui Zhang, Shi-Wen Jiang, Qun Fang, Jia-Xu Chen, Xiao-Nong Zhou

**Affiliations:** 1 National Institute of Parasitic Diseases, Chinese Center for Disease Control and Prevention, Key Laboratory of Parasite and Vector Biology, Ministry of Health, WHO Collaborating Centre for Malaria, Schistosomiasis and Filariasis, 207 Rui Jin Er Road, Huangpu District, Shanghai 200025, P. R. China; 2 National Center for Tuberculosis Control and Prevention, Chinese Center for Disease Control and Prevention, 155 Changbai Road, Changping District, Beijing 102206, P. R. China

**Keywords:** Pulmonary tuberculosis, Spatial variations, Impact factor, Cokriging, China

## Abstract

**Background:**

The report of the fifth national tuberculosis (TB) epidemiological survey in P. R. China, 2010, roughly showed that pulmonary TB (PTB) prevalence was higher in western China than in central and eastern China. However, accurately estimating the continuous spatial variations of PTB prevalence and clearly understanding factors impacting on spatial variations of PTB prevalence are important for allocating limited resources of national TB programme (NTP) in P. R. China.

**Methods:**

Using ArcGIS Geostatistical Wizard (ESRI, Redlands, CA), an evaluation was performed to decide that which kriging and cokriging methods along with different combinations of types of detrending, semivariogram models, anisotropy and covariables (socio-economic and geographic factors) can accurately construct spatial distribution surface of PTB prevalence using statistic data sampled from the fifth national TB epidemiological survey in P. R. China, 2010, and then the evaluation results were used to explore factors of spatial variations.

**Results:**

The global cokriging with socio-economic and geographic factors as covariables proved to be the best geostatistical methods for accurately estimating spatial distribution surface of PTB prevalence. The final continuous surfaces of PTB prevalence distribution demonstrated that PTB prevalence were lower in Beijing, Tianjin, Shanghai and southeastern coast China, higher in western and southwestern China, and crossed between low and high in central China.

**Conclusions:**

The predicted continuous surface perspicuously illustrated the spatial variations of PTB prevalence that were co-impacted by socio-economic and geographic factors, which can be used to better allocate the always limited resources of NTP in P. R. China.

## Background

In 2010, Disease Control Bureau of the Ministry of Health, People’s Republic of China (P. R. China) and Chinese Center for Disease Control and Prevention implemented the fifth national tuberculosis (TB) epidemiological survey, due to logistical and financial limitations, which only was conducted through sampling a limited number of point locations throughout the country and roughly found that the active, *Mycobacterium* positive and smear positive pulmonary TB (PTB) prevalence was higher in western China than in central and eastern China [1]. However, what factors have significant impacts on these spatial variations of PTB prevalence are not quite clear in P. R. China. Accurately estimating the continuous surface of TB prevalence and clearly understanding factors of spatial variations are important for allocating limited resources of national TB programme (NTP) and prioritizing the areas with serious TB prevalence relative to another. Therefore, it is necessary to understand the patterns on spatial heterogeneity of PTB prevalence using statistic data sampled from the fifth national TB epidemiological survey and explore factors of spatial heterogeneity in P. R. China.

In order to understand the patterns on spatial heterogeneity, some types of spatial data analysis method could be used to estimate data values at unobserved locations from observation of its value at nearby locations. Generally, most of the studies in spatial data analysis can be divided into two branches: the model-driven approach, e.g. spatial regression analysis, and the data-driven approach, e.g. kriging methods. A study found that kriging provide the smaller error measures than multiple linear regression model, spatial lag model and spatial error model, so it was concluded that kriging has a clear advantage for spatial data analysis compared to spatial regression analysis [2].

Kriging is one of interpolation methods, which apply regionalized variables and describe spatial dependencies between the instances of random variables by using semivariograms [3]. A semivariogram is a graphical display of a variance of measurements over the distance between the measurement sites. If there are spatial dependencies the variance between the observations on two points normally increases with increasing distance until at a specific range a maximum value is reached. Considered to be the most sophisticated geostatistical method, kriging can potentially provide the most accurate results of continuous surface estimates, and has been more and more often used for epidemiological mapping of infectious disease, such as TB [4], schistosomiasis [5], malaria [6], cholera [7], dysentery [7] and influenza-like illness [8]. However, kriging is applied narrowly in discipline of TB control and prevention in P. R. China.

Unlike kriging, which only use data available at the target location and fail to use existing spatial correlations from secondary-data control points and the primary attribute to be estimated, cokriging not only requires the same conditions to be satisfied as kriging does, but also can take advantage of the covariance between two or more regionalized variables that are related, which proved to be beneficial to better estimate map values in a study [9]. As an important public health problem, TB prevalence has been influenced by not only socio-economic factors but also geographic factors worldwide. For example, a study in Brazil showed that TB incidence and socio-economic status had a significant curvilinear relationship [10], and another study in Mexico found that altitude had a strong inverse relationship to PTB notification rates [11]. However, it is not clear whether socio-economic and geographic attributes can impact on TB prevalence, and compared with kriging, cokriging along with these factors as covariables can improve accuracies of continuous surface estimate about TB prevalence in P. R. China.

In this study, using the dataset of the fifth national TB epidemiological survey in 2010 [1], kriging and cokriging along with different trend removal, anisotropy, semivariogram models and cokriging combined with information on socio-economic and geographic attributes were performed to find the appropriate methods that can provide most accurate distribution estimates of PTB prevalence in P. R. China. Based on the appropriate methods, socio-economic and geographic factors impacting on spatial variations of PTB prevalence were evaluated, and spatial distribution of PTB prevalence were generated, which can be helpful to allocating limited resources of NTP in P. R. China.

## Methods

### Data sources of TB prevalence

**Figure 1:**
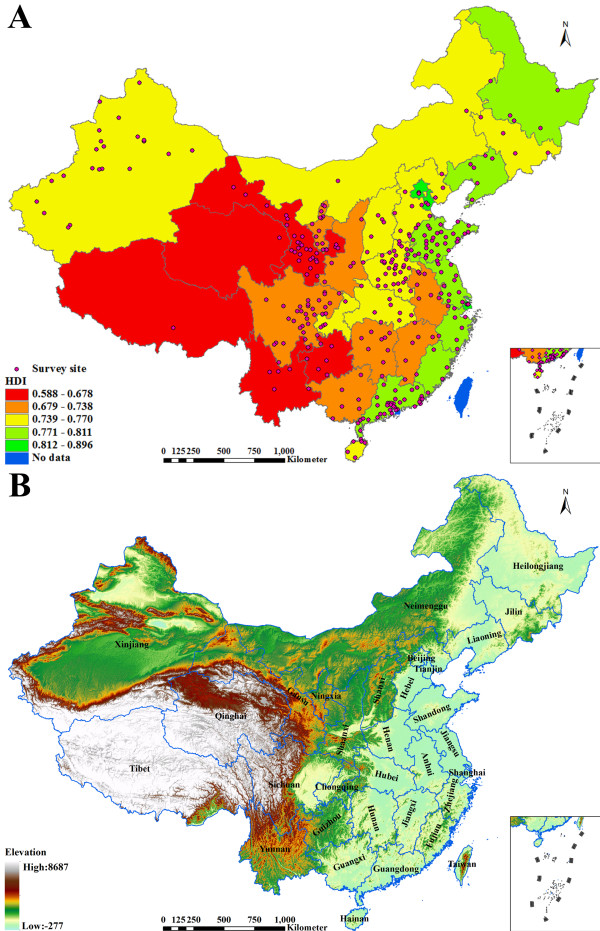
Location of survey sites, human development index (HDI), and elevation (A: distributions of survey sites and HDI; B: map of the digital elevation model).

The dataset of TB prevalence was obtained from the fifth national TB epidemiological survey in 2010, which included active PTB prevalence, sputum *Mycobacterium* positive PTB prevalence and sputum smear positive PTB prevalence. Active PTB includes smear positive PTB, smear negative PTB and tuberculous pleurisy, and *Mycobacterium* positive PTB includes smear positive PTB and smear negative PTB with culture positive [12]. Multi-stage stratified cluster random sampling, with the probability proportionate to population size, was used to select 176 survey sites across the country, and about 1500 subjects were surveyed in each survey site [1], which were called survey sites of national level. Additionally, except survey sites of national level , Shandong, Henan, Guangdong, Hainan, Sichuan, Gansu, Ningxia and Xinjiang sampled the survey sites again in their provinces respectively, according to the national sampling methods, and they totally sampled 151 survey sites [13], which were called survey sites of provincial level. Total 327 survey sites including national level and provincial level as well as supporting data were converted into a Geodatabase format of Environmental Systems Research Institute (ESRI) for calculating in this study (Figure 1A).

### Origin of socio-economic covariable

Human development index (HDI) was used as the socio-economic covariable for cokriging in this study, which is a composite statistic of life expectancy, education, and income indices used to reflect human development, well-being concept based on capability approach, published by the United Nations Development Programme [14]. By concentrating on aspects beyond income and treating income as a proxy for a decent standard of living, the HDI provides a more comprehensive picture of human life than income only [14]. So the HDI is the appropriate indicator representing the socio-economic attributes. The HDIs by province in P. R. China, 1999, 2003, 2005 and 2008 were collected [15-18], and the data values of 4 years were averaged by province to increase the stability of data and minimized the bias, which were converted into an ESRI Geodatabase format for calculating in this study. Figure 1A showed the averaged values of HDI across the country.

### Origin of geographic covariable

The Digital Elevation Model (DEM) was used as the geogrphic covariable for cokriging in this study, which has a spatial resolution of 200 m, and was obtained from the website of Data Sharing Infrastructure of Earth System Science (http://www.geodata.cn). Figure 1B illustrated the elevational gradients of whole country in P. R. China. It was proved that the elevation, as one of the geographic attributes, has close correlations with TB prevalence in many countries, such as in Mexico, Kenya, Peru and Turkey [11,19-22]. Therefore, the elevation was considered as the better covariable to estimate TB prevalence. All digital datasets including TB prevalence of survey sites, HDIs by province and DEM were transformed to the same cartographic projection.

### Testing of kriging and cokriging

**Figure 2:**
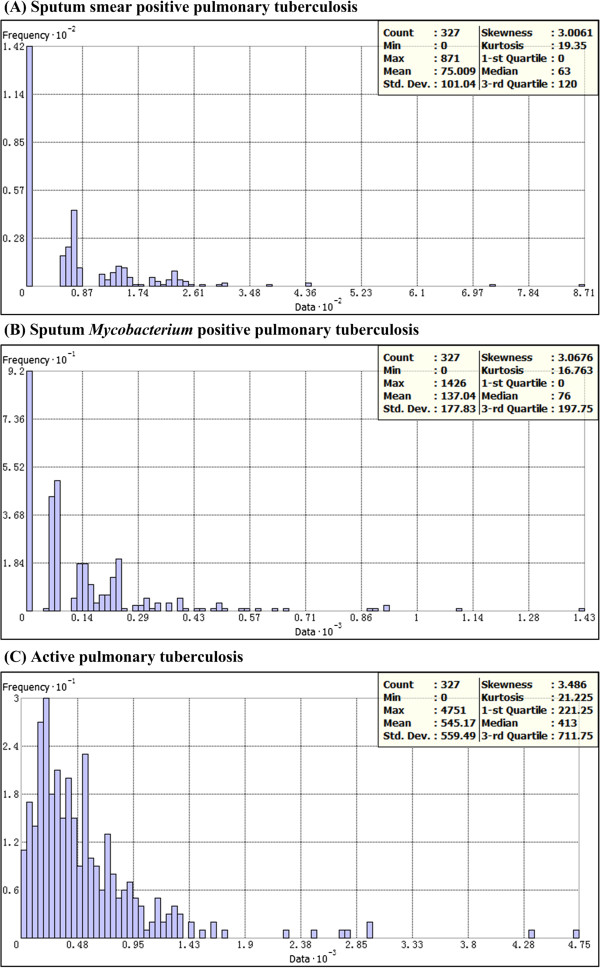
**Histogram of pulmonary tuberculosis (PTB) prevalence in survey sites (A: sputum smear positive PTB; B: sputum *Mycobacterium *** **positive PTB; C: active PTB).**

**Figure 3:**
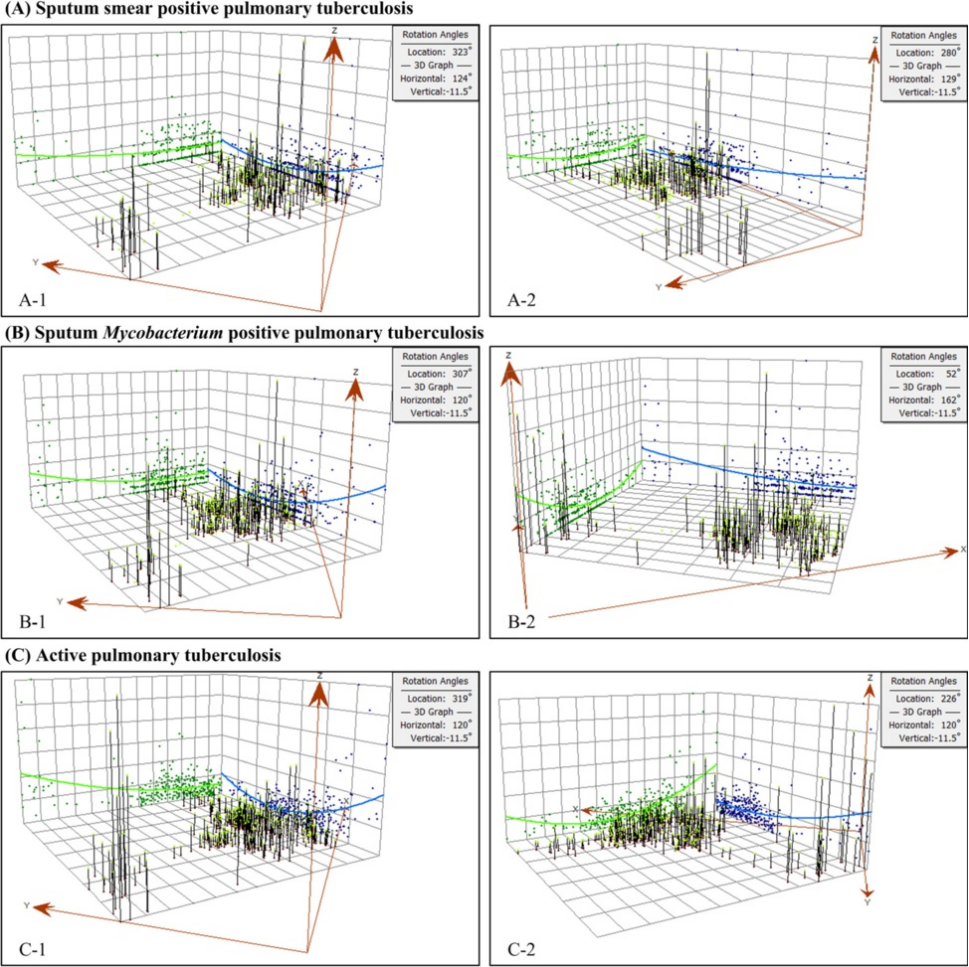
**Trend analysis of pulmonary tuberculosis (PTB) prevalence in survey sites (A: sputum smear positive PTB; B: sputum *Mycobacterium *** **positive PTB; C: active PTB).**

Given that smear positive PTB prevalence, *Mycobacterium* positive PTB prevalence and active PTB prevalence in survey sites presented skew distributions (Figure 2) and U-shaped curves in different directions (Figure 3), it was difficult to choose a clear, univocal geostatistical method along with geostatistical algorithm most accurately estimating PTB prevalence distribution. An evaluation was performed to decide that which geostatistical methods (i.e., ordinary kriging and ordinary cokriging) along with types of detrending (i.e., global, neighborhood and local), semivariogram models (i.e., circular, spherical, tetraspherical, pentaspherical, exponential, Gaussian, rational quadratic, hole effect, K-Bessel, J-Bessel and stable), anisotropy (i.e., true and false) and covariables (i.e., HDI and elevation) would provide the most accurate estimation of PTB prevalence surface. For each class of PTB prevalence, totally 264 geostatistical methods of data interpolation were applied and compared (Additional files 1, 2 and 3).

The geostatistical method that was selected to generate maps of PTB prevalence distribution was based on statistical characteristics of each output surface based on comparison of cross-validation measures [23]. Four cross-validation prediction error parameters were taken into account: root-mean-square (RMS), mean standardized (MeanStan), root-mean-square standardized (RMSStan) and average standard errors (ASE) for geostatistical methods. A better geostatistical method satisfies the following conditions at the same time: RMS is smaller, MeanStan is nearly 0, RMSStan is nearly 1, and ASE approaches RMS.

In order to comprehensively utilize these parameters to provide a better geostatistical method, value of RMS, absolute value of MeanStan, value of RMSStan and difference value of subtracting ASE from RMS were sorted in ascending order in total methods, respectively, and then their ranks were summed up for each method (Additional files 1, 2 and 3). Based on the lowest prediction parameters error criteria, the method having the smallest total ranks indicated the best geostatistical method. Table 1 showed evaluation results of top 10 best geostatistical methods for each class of PTB prevalence.

**Table 1:** Evaluation of ordinary kriging and ordinary cokriging with various combinatorial approaches (evaluation results of top 10 best methods for each class of PTB prevalence)

**Variable**	**Covariable**	**Type of detrending**	**Anisotropy**	**Model**	**Prediction errors**					**Rank**				
					**Mean**	**RMS**	**Meanstan**	**RMSStan**	**ASE**	**RMS**	**AbsMeanStan**	**RMSStan**	**RMSASE**	**Total**
Smear positive PTB prevalence	HDI + Elevation	Global	TRUE	K-Bessel	0.07657	94.16	0.0006996	1.031	91.36	44.5	14.0	14.5	15.5	88.5
	HDI + Elevation	Neighborhood	TRUE	K-Bessel	−0.02200	94.12	−0.0002937	1.037	90.83	40.0	6.0	46.5	43.5	136.0
	HDI + Elevation	Local	TRUE	Stable	−0.08795	93.82	−0.0009434	1.038	90.47	15.5	21.0	53.0	49.5	139.0
	HDI + Elevation	Local	TRUE	Gaussian	−0.12560	93.95	−0.0013150	1.037	90.63	29.0	36.0	46.5	48.0	159.5
	HDI	Local	TRUE	Pentaspherical	0.05834	93.54	0.0006271	1.044	89.66	1.0	12.0	79.0	76.0	168.0
	HDI	Global	TRUE	Stable	0.27480	94.02	0.0027300	1.028	91.52	34.0	122.0	10.0	9.0	175.0
	HDI	Neighborhood	TRUE	Stable	0.24590	93.87	0.0025130	1.033	90.94	22.5	110.0	22.5	23.0	178.0
	HDI + Elevation	Neighborhood	TRUE	Gaussian	0.05429	94.30	0.0005347	1.038	90.88	68.0	10.0	53.0	55.0	186.0
	HDI + Elevation	Local	TRUE	Pentaspherical	0.19470	93.57	0.0021250	1.039	90.08	2.5	78.0	56.5	59.0	196.0
	HDI + Elevation	Global	TRUE	Stable	0.28060	94.13	0.0028940	1.031	91.33	41.5	131.0	14.5	15.5	202.5
*Mycobacterium* positive PTB prevalence	HDI + Elevation	Global	TRUE	J-Bessel	0.87820	151.0	0.00587800	1.089	138.2	8.0	105.0	1.0	1.0	115.0
		Global	TRUE	Stable	0.24530	152.7	0.00135700	1.100	138.5	139.0	16.0	12.5	11.0	178.5
	HDI	Global	TRUE	J-Bessel	0.25230	152.6	0.00124300	1.107	137.4	127.5	13.0	28.0	28.0	196.5
	Elevation	Global	TRUE	Hole Effect	0.84340	152.3	0.00561100	1.094	138.8	103.0	95.0	2.0	2.0	202.0
	HDI	Global	TRUE	Hole Effect	0.07334	152.8	−0.00008275	1.106	137.7	149.0	1.0	27.0	27.0	204.0
	Elevation	Global	TRUE	J-Bessel	0.85340	152.5	0.00544800	1.099	138.4	117.5	83.0	9.5	8.5	218.5
	Elevation	Global	TRUE	K-Bessel	0.46630	153.0	0.00294700	1.101	138.5	184.5	31.0	15.5	16.5	247.5
		Neighborhood	TRUE	Stable	0.32670	152.7	0.00200600	1.117	136.3	139.0	20.0	48.5	49.0	256.5
	HDI	Neighborhood	TRUE	J-Bessel	0.50320	152.3	0.00318000	1.121	135.4	103.0	37.0	64.0	63.5	267.5
	HDI + Elevation	Neighborhood	TRUE	J-Bessel	1.27800	151.5	0.00886400	1.110	136.1	21.0	197.0	32.5	30.0	280.5
Active PTB prevalence	HDI + Elevation	Global	FALSE	Pentaspherical	0.5921	426.5	0.003456	1.255	333.5	28.5	55.0	44.0	54.5	182.0
	HDI	Global	TRUE	Gaussian	0.9281	432.5	0.002311	1.235	348.0	156.5	27.0	7.0	6.0	196.5
	HDI + Elevation	Global	FALSE	Tetraspherical	0.8312	426.8	0.004003	1.251	335.2	39.0	76.0	38.5	46.5	200.0
	HDI	Global	TRUE	K-Bessel	1.1140	432.0	0.002998	1.236	347.3	144.5	42.0	9.0	7.5	203.0
	HDI	Global	TRUE	Hole Effect	0.4762	433.4	0.001026	1.240	347.2	171.5	7.0	14.5	13.5	206.5
		Global	FALSE	Tetraspherical	0.5214	426.5	0.003206	1.261	331.9	28.5	49.0	55.5	73.5	206.5
	HDI	Global	FALSE	Tetraspherical	0.5249	426.5	0.003222	1.261	331.9	28.5	50.0	55.5	73.5	207.5
	HDI + Elevation	Global	FALSE	Spherical	1.1160	427.2	0.004647	1.247	336.8	48.5	90.0	30.0	39.0	207.5
		Global	FALSE	Pentaspherical	0.2991	426.2	0.002701	1.265	330.4	23.0	32.0	70.5	83.0	208.5
		Global	FALSE	Spherical	0.7560	426.8	0.003742	1.257	333.4	39.0	65.0	46.0	59.5	209.5

PTB, pulmonary tuberculosis; HDI, human development index; RMS, root-mean-square; MeanStan, mean standardized; RMSStan, root-mean-square standardized; ASE, average standard errors; AbsMeanStan, absolute value of MeanStan; RMSASE, difference value of subtracting ASE from RMS.

### Geostatistics software

Maps showing spatial distribution prediction of PTB prevalence and prediction standard errors that shows the uncertainty related to the predicted values were created with the Geostatistical Wizard to ArcGIS (ArcGIS 10; ESRI Inc., Redlands, CA, USA), and the Natural Breaks (Jenks) method was used to classify the predicted values and their standard errors. ArcGIS also was used to convert datasets without Geodatabase format into an ESRI Geodatabase format, transform all digital datasets to the same cartographic projection, and evaluate geostatistical methods with different parameters combination. Except types of detrending, types of semivariogram models, anisotropy and covariables, other parameters (nugget, partial sill, etc.) of kriging and cokriging were estimated using an iterative cross validation technique to optimize semivariogram models in ArcGIS.

## Results

### Results of cross-validation

**Figure 4:**
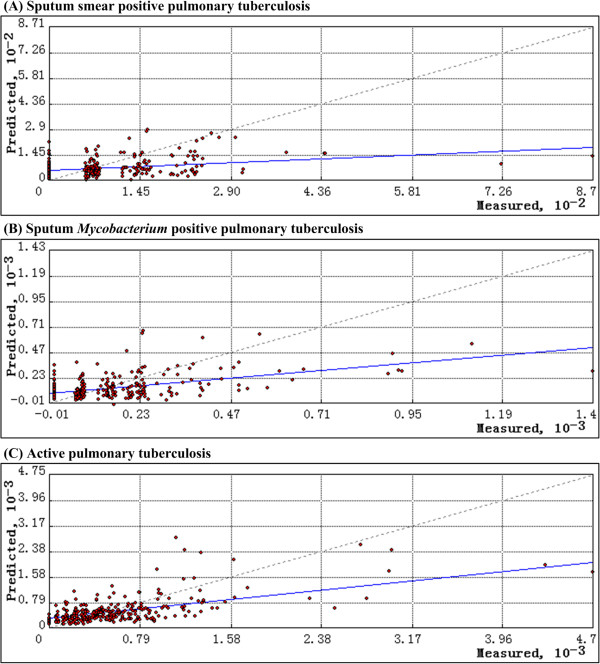
**Scatterplot of predicted values versus measured values in the geostatistical method finally selected for continuous surface estimation of pulmonary tuberculosis (PTB) prevalence (A: sputum smear positive PTB; B: sputum *Mycobacterium *** **positive PTB; C: active PTB).**

**Figure 5:**
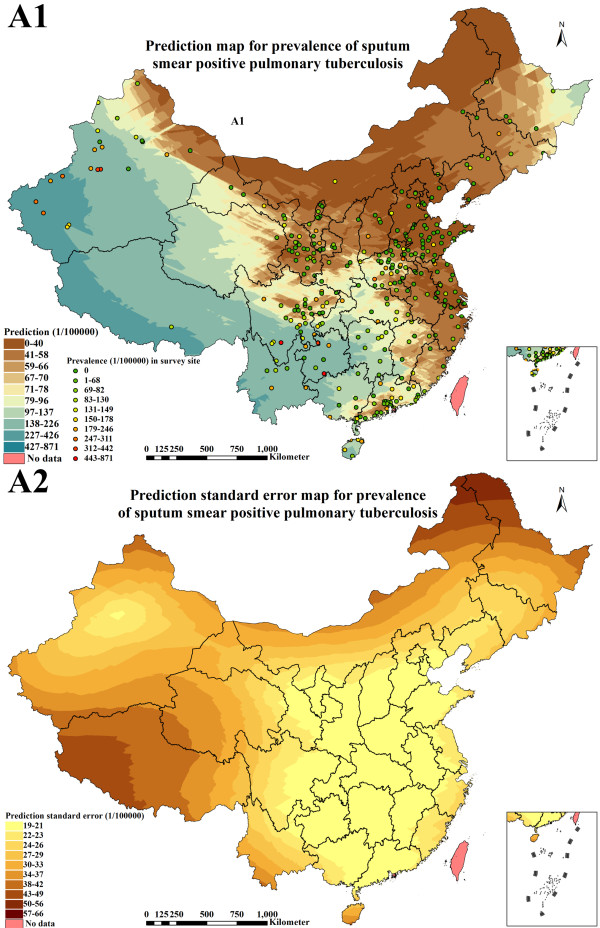
Prediction map (1 × 1 km spatial resolution) and prediction standard error map (1 × 1 km spatial resolution) created with the geostatistical method finally selected for continuous surface estimation of sputum smear positive pulmonary tuberculosis prevalence (A1: prediction; A2: prediction standard error).

**Figure 6:**
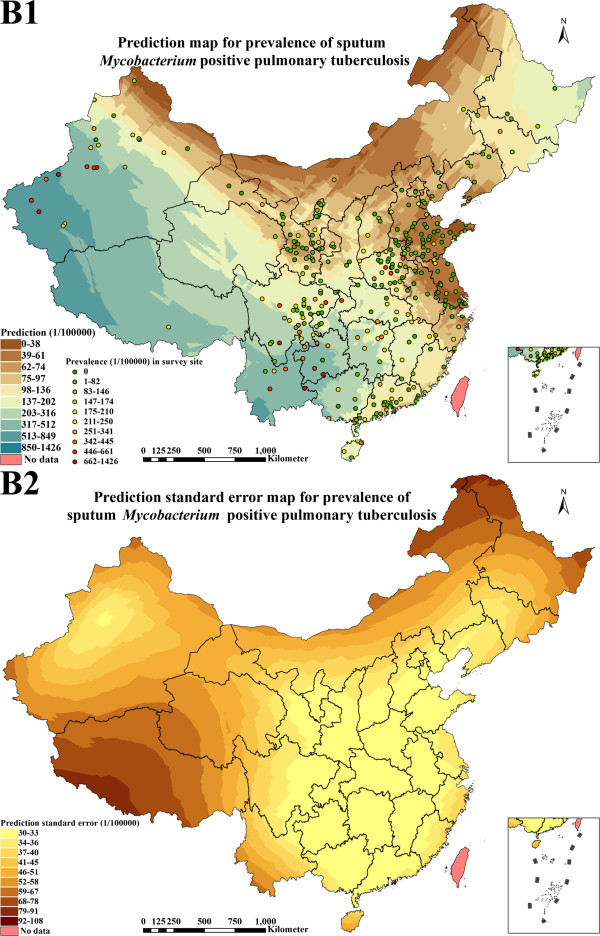
**Prediction map (1 × 1 km spatial resolution) and prediction standard error map (1 × 1 km spatial resolution) created with the geostatistical method finally selected for continuous surface estimation of sputum *Mycobacterium *** **positive pulmonary tuberculosis prevalence (B1: prediction; B2: prediction standard error).**

**Figure 7:**
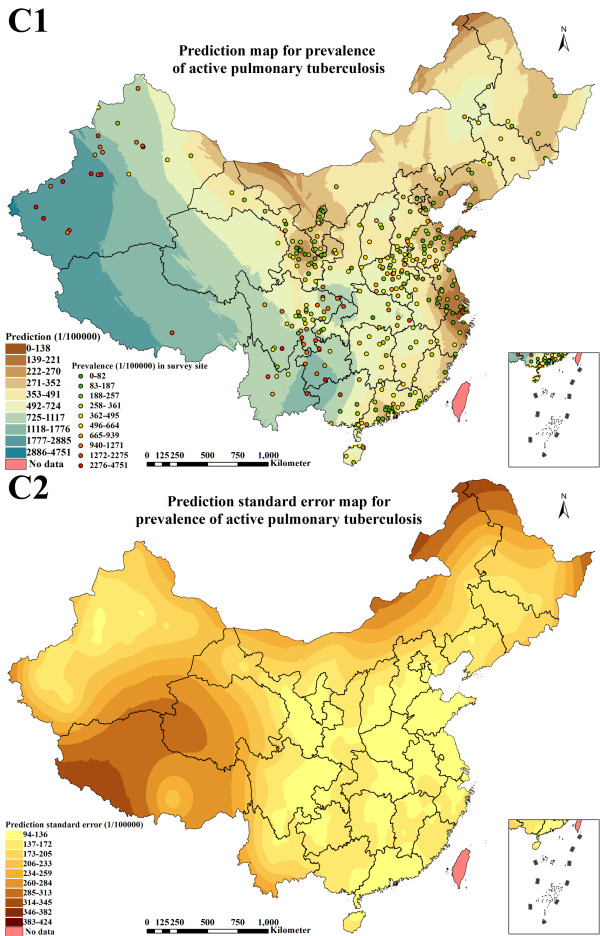
Prediction map (1 × 1 km spatial resolution) and prediction standard error map (1 × 1 km spatial resolution) created with the geostatistical method finally selected for continuous surface estimation of active pulmonary tuberculosis prevalence (C1: prediction; C2: prediction standard error).

For smear positive PTB prevalence, the best geostatistical method was K-Bessel model of ordinary cokriging with global detrending, with true anisotropy and with HDI and elevation as covariables. For *Mycobacterium* positive PTB prevalence, the best one was J-Bessel model of ordinary cokriging with global detrending, with true anisotropy and with HDI and elevation as covariables. For active PTB prevalence, the best one was pentaspherical model of ordinary cokriging with global detrending, with false anisotropy and with HDI and elevation as covariables (Table 1). Figure 4 suggested that larger measured values tended to be underpredicted and smaller measured values tended to be overpredicted in the best geostatistcal method for estimating the distribution of each class of PTB prevalence, which is a property of kriging and cokriging [24]. Figures 5, 6 and 7 showed that the uncertainty of predicted values in the border of Heilongjiang and Neimenggu, Tibet and western Qinghai were larger than in other areas.

### Distribution estimate of smear positive PTB prevalence

Figure 5 illustrated smear positive PTB prevalence prediction map (1 × 1 km spatial resolution) and prediction standard error map (1 × 1 km spatial resolution) according to the best geostatistical method. The range of the prevalence was 0 to 426 per 100,000 population in P. R. China, in which the predicted values increased by degrees in eastern, central and western China but presented interlocked distributions in some pockets of the country. The prevalence (0 to 70 per 100,000 population) in Beijing, Tianjin, Hebei, Shanxi, Neimenggu, Liaoning, Shanghai, Jiangsu, Zhejiang, Anhui, Fujian, Shandong and Ningxia were relatively lower than in other provinces. The prevalence in Jilin, Heilongjiang, Jiangxi, Henan, Hubei, Guangdong, northern Sichuan, Shaanxi, Gansu, eastern Qinghai and northern Xinjiang presented interlocked distributions between 0 and 137 per 100,000 population. In Hunan, Guangxi, Hainan, Chongqing, southern Sichuan, Guizhou, Yunnan, Tibet, western Qinghai and southern Xinjiang, the prevalence increased gradually from 97 to 426 per 100,000 population.

### Distribution estimate of *Mycobacterium* positive PTB prevalence

Figure 6 illustrated *Mycobacterium* positive PTB prevalence prediction map (1 × 1 km spatial resolution) and prediction standard error map (1 × 1 km spatial resolution) according to the best geostatistical method. The range of the prevalence was 0 to 849 per 100,000 population in P. R. China, in which the predicted values increased by degrees in eastern, central and western China but presented interlocked distributions in some pockets of the country. The prevalence (0 to 136 per 100,000 population) in Beijing, Tianjin, Hebei, Shanxi, Neimenggu, Liaoning, Shanghai, Jiangsu, Zhejiang, Anhui, Fujian, Shandong and Ningxia were relatively lower than in other provinces. The prevalence in Jilin, Heilongjiang, Jiangxi, Henan, Hubei, Hunan, Guangdong, Guangxi, Hainan, Chongqing, Sichuan, Shaanxi, Gansu, Qinghai and northern Xinjiang presented interlocked distributions between 0 and 512 per 100,000 population. In Guizhou, Yunnan, Tibet and southern Xinjiang, the prevalence increased gradually from 317 to 849 per 100,000 population.

### Distribution estimate of active PTB prevalence

Figure 7 illustrated active PTB prevalence prediction map (1 × 1 km spatial resolution) and prediction standard error map (1 × 1 km spatial resolution) according to the best geostatistical method. The range of the prevalence was 0 to 4,751 per 100,000 population in P. R. China, in which the predicted values increased by degrees in eastern, central and western China but presented interlocked distributions in some pockets of the country. The prevalence (0 to 491 per 100,000 population) in Beijing, Tianjin, Hebei, Liaoning, Shanghai, Jiangsu, Zhejiang, Fujian, Shandong and Ningxia were relatively lower than in other provinces. The prevalence in Shanxi, Neimenggu, Jilin, Heilongjiang, Anhui, Jiangxi, Henan, Hubei, Hunan, Guangdong, eastern Guangxi, Hainan, Chongqing, northern Sichuan, Shaanxi, Gansu, eastern Qinghai and northern Xinjiang presented interlocked distributions between 0 and 1,117 per 100,000 population. In western Guangxi, southern Sichuan, Guizhou, Yunnan, Tibet, western Qinghai and southern Xinjiang, the prevalence increased gradually from 725 to 4,751 per 100,000 population.

## Discussion

Obtaining an accurate prediction is the ultimate aim of most studies that use kriging or cokriging. To improve the accuracy, many studies always selected a kriging or cokriging method they thought fit, or compared two or more kriging or cokriging methods to find the fittest one [5-8,25]. However, it is difficult to find the best fitness method that can provide the most accurate prediction because four cross-validation prediction error parameters can hardly meet requires at the same time in a method when many methods are compared. To solve this problem, we developed a comprehensive determination criterion in this study, which rapidly determined the comprehensive positions of four cross-validation prediction error parameters meeting requires at the same time in 264 combinations of geostatistical input parameters for both kriging and cokriging for each class of PTB prevalence. Therefore, we had good reasons to believe that the final cokriging methods selected in this study ensured considerable accuracy of spatial prediction because we had compared most methods in a study so far.

Results of cross-validation in this study showed that global cokriging with HDI and elevation as covariables was the best geostatistical methods, which suggested that HDI and elevation as covariables increased the accuracy of spatial prediction for TB prevalence. In deeper order, this reflects that socio-economic factors and geographical factors can affect TB prevalence in P. R. China, which confirmed our hypotheses according to previous studies conducted in other countries [10,11,19-22,26-28]. Therefore, except adopting socio-economic measures to control and prevent TB in P. R. China, impacts of geographic factors on TB control and prevention should be evaluated and interventions according with geographic features also should be adopted.

Continuous surfaces estimation of PTB prevalence in this study demonstrated that sputum smear positive, sputum *Mycobacterium* positive and active PTB prevalence were lower in Beijing, Tianjin, Shanghai and southeastern coast China, and were higher in western and southwestern China, which was consistent with the report on the fifth national TB epidemiological survey [1]. However, distributions of PTB prevalence were complex in central China, which presented interlocked distributions between low and high PTB prevalence. This situation would increase complexities and difficulties of TB control and prevention in these areas, which would slow down the progress of NTP, given that 53% of the total population in the country is in these areas [29]. Consequently, in order to achieve the goal of NTP according to schedule, on the basis of keeping the current level in eastern China and strengthening the further effort in western China, central China should be as the prior areas of TB control and prevention.

Although we thought that spatial prediction of PTB prevalence was considerably accurate in this study, we found that the uncertainty of predicted values in the border of Heilongjiang and Neimenggu, Tibet and western Qinghai were larger than in other areas. It was obvious that survey sites were sparser in areas with higher uncertainty of predicted values. Guimaraes, et al. [5] advised that, to improve the accuracy of an estimate using kriging, it would be necessary to obtain data with better location and spatial distribution of the information collected in the fieldwork. However, the probability proportionate to population size was merely considered when sampling survey sites in the fifth national TB epidemiological survey in P. R. China, which led to that survey sites were sparser in the vast, sparsely populated areas [1]. Therefore, if we hope to obtain accurate and stable surface estimate through sampling survey in P. R. China in the future, we need to consider not only proportion of population when sampling survey sites but also their rational spatial distribution.

## Conclusion

In conclusion, cokriging proved to be a suitable tool for accurately estimating the continuous surface of TB prevalence in P. R. China when socio-economic and geographic factors were considered as covariables, which suggested that these factors had impacts on regional differences of TB prevalence. The predicted surface of TB prevalence perspicuously demonstrated that sputum smear positive, sputum *Mycobacterium* positive and active PTB prevalence were lower in Beijing, Tianjin, Shanghai and southeastern coast China, higher in western and southwestern China, and crossed between low and high in central China. These findings can be used to better allocate the always limited resources of NTP.

## Competing interests

We the authors declare that we have no competing interests.

## Authors’ contributions

X-X L, L-X W and X-N Z conceived and designed the study; H Z, S-W J and Q F contributed materials, X-X L analyzed the data and wrote the first draft of the manuscript, L-X W and J-X C provided constructive opinions and suggestions, X-N Z provided strategic advices and assisted with editing of the manuscript. All authors read and approved the final version of the manuscript.

## Pre-publication history

The pre-publication history for this paper can be accessed here:

http://www.biomedcentral.com/1471-2458/14/257/prepub

## Supplementary Material

Additional file 1 Evaluation of ordinary kriging and ordinary cokriging with various combinatorial approaches for smear positive PTB prevalence. Click here for file

Additional file 2 **Evaluation of ordinary kriging and ordinary cokriging with various combinatorial approaches for *Mycobacterium *** **positive PTB prevalence.** Click here for file

Additional file 3 Evaluation of ordinary kriging and ordinary cokriging with various combinatorial approaches for active PTB prevalence. Click here for file
